# A Misdiagnosed Odontogenic Tumor: A Clinical Dilemma

**DOI:** 10.5005/jp-journals-10005-1436

**Published:** 2017-06-01

**Authors:** Chitrita G Mukherjee, Uday Mukherjee, Anju Bansal, Anupriya Jha

**Affiliations:** 1 Professor and Head, Department of Pediatric and Preventive Dentistry, Buddha Institute of Dental Sciences and Hospital, Magadh University Bodh Gaya, Bihar, India; 2 Professor and Head, Department of Dentistry, KPC Medical College and Hospital Kolkata, West Bengal, India; 3 Reader, Department of Pedodontics and Preventive Dentistry, Buddha Institute of Dental Sciences and Hospital, Patna, Bihar, India; 4 Postgraduate Student, Department of Pedodontics and Preventive Dentistry, Buddha Institute of Dental Sciences and Hospital, Patna, Bihar, India

**Keywords:** Ameloblastomas, Myxomas, Odontogenic cysts.

## Abstract

Odontogenic myxomas are rarely occurring, slow-growing, asymptomatic, and locally aggressive odontogenic tumors with high rate of recurrence, mainly of ectomesenchymal origin. Three-dimensional imaging techniques can be used to diagnose such pathological lesions, but have limited use as they are not cost-effective and are selectively available for better outcome. Following is a case report of OM of a 5-year-old child with a brief discussion on its diagnosis and management.

**How to cite this article:**

Mukherjee CG, Mukherjee U, Bansal A, Jha A. A Misdiagnosed Odontogenic Tumor: A Clinical Dilemma. Int J Clin Pediatr Dent 2017;10(2):205-207.

## INTRODUCTION

An odontogenic myxoma (OM) is a rare, benign, and locally aggressive odontogenic tumor characterized by gross replacement of cancellous bone by gelatinous or mucoid tissue, thus leading to cortical bone expansion. It originates from the embryonic mesenchymal elements of the developing tooth. The World Health Organization has categorized OM as a benign tumor of ectomesenchymal origin with or without the presence of odontogenic epithelium.^1^

They are slow-progressing, asymptomatic, and site-aggressive tumors. The lesions may reach a questionable size before the patient realizes its existence and seeks treatment. Odontogenic myxoma is not easy to diagnose since it has variable histopathological and radiological features.

In this study, we present a case of central OM of mandible with a brief review of the clinical, radiological, and histopathological features.

## CASE REPORT

**Fig. 1:**
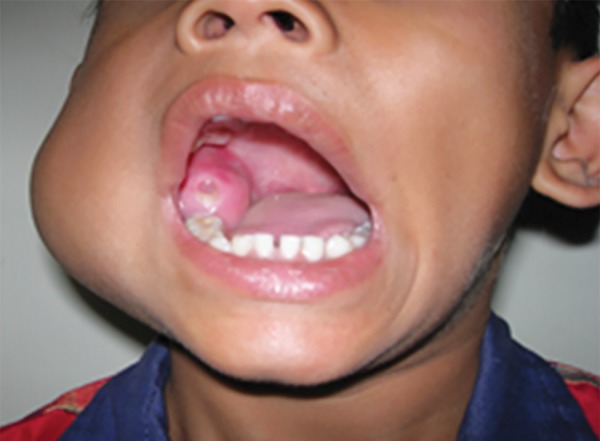
Bony hard, non- tender swelling inside the oral cavity,distal to the deciduous second molar

**Fig. 2:**
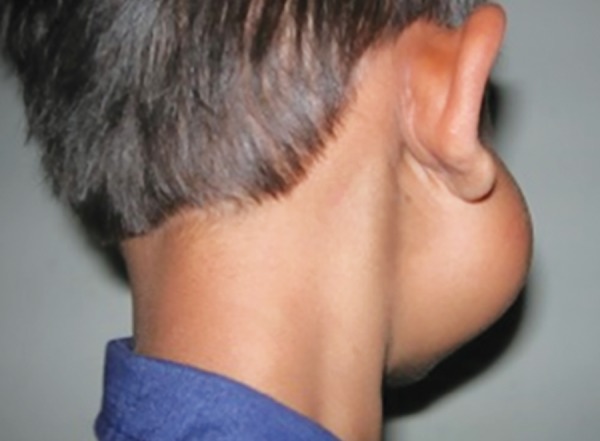
Swelling, extending superoinferiorly from the posterior border of mandible upto tragus of the right ear

A 5-year-old otherwise healthy boy presented with a slowly enlarging painless swelling on the lower right side of his face. The clinical examination revealed he had a bony hard, nontender swelling, extending from the posterior border of the mandible up to tragus of the right ear, while the overlying skin was normal in color without any secondary changes (Figs 1 and 2).

**Fig. 3:**
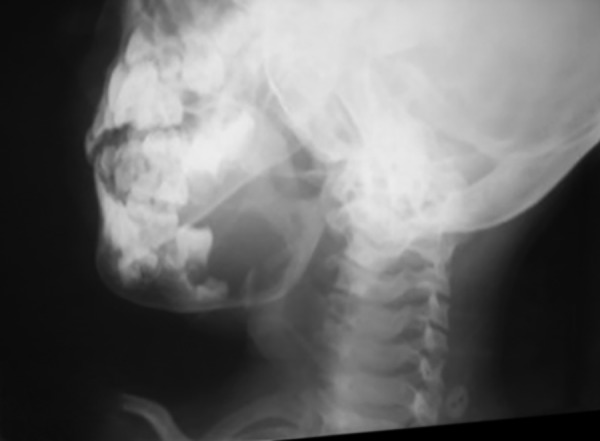
The lateral oblique radiograph of the jaw

**Fig. 4:**
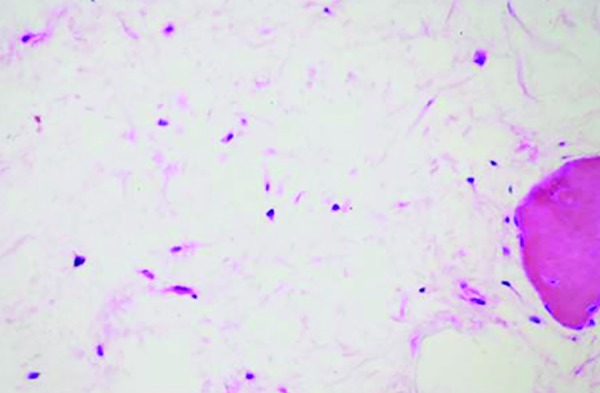
Photomicrograph shows randomly arranged stellate cells in a loose myxoid stroma

**Fig. 5:**
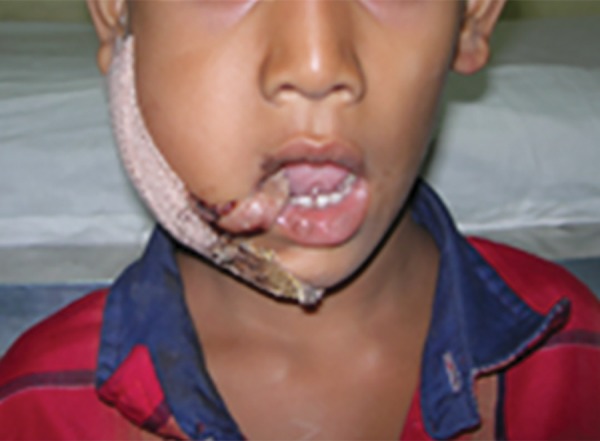
Postoperative

The lateral oblique radiograph of the jaw revealed a unilocular radiolucent lesion extending from the distal surface of the first permanent mandibular molar, involving almost the entire ramus encapsulating an unerupted tooth (Fig. 3), with intact lower/inferior border of the mandible.

On aspiration, as there was no cystic fluid available, provisional diagnosis of unilocular ameloblastoma was made. Routine blood investigation was done with no abnormal findings. Complete removal of lesion was planned under general anesthesia after obtaining a written consent and a thorough preanesthetic evaluation.

The tumor was exposed intraorally with a midcrestal incision. With adequate exposure, the junction of normal bone and pathological tumor was identified, followed by thorough surgical curettage of the lesion. The lower border of mandible was intact and utmost care was taken to prevent pathological fracture. Since the growth and development of the mandible may be affected, bone plating was avoided.

A solid tumor with an impregnated tooth approximately measuring 4 × 5 cm and the entire tumor were sent for histopathological evaluation.

Hematoxylin and eosin staining (100x) revealed sheets of stellate-shaped cells with long anastomosing processes, scattered in mildly basophilic collagenous stroma along with a few strands of odontogenic epithelium, and foci of calcification was also found (Fig. 4).

The overall features were suggestive of central OM. The patient was recalled at regular intervals for clinical and radiographic examinations to monitor the healing and recurrence of the lesion (Fig. 5).

## DISCUSSION

Prevalence of OM is between 0.04 and 3.7%,^2^ commonly seen in the age group between 10 and 50 years, OM affects mandible more often than maxilla.^1^

Odontogenic myxoma has been classified based on radiographic appearances into six types by Zhang et al:^3^ Type I: Unilocular well-defined radiolucency; type II (multilocular): two or more compartments with multiple interlaced osseous trabeculae described as honey comb, soap bubble, or tennis racquet radiolucency; type III: lesion located in alveolar bone; type IV: lesion involving the maxillary sinus; type V (moth-eaten appearance): larger radiolucent area with irregular borders; type VI: combination of bone destruction and bone formation giving sun ray appearance. The tumor often shows scalloping between the roots; root resorption can occur but is rare.^4^ The abovementioned case is type I OM.

Odontogenic myxomas have uncertain pathogenesis. There are two school of thoughts, one of which says that OM is a tumor arising from mesenchymal portion of tooth germ, either from the dental papilla or follicle. Absolute proof of origin from odontogenic apparatus is lacking, but it is most likely due to its frequent occurrence in jaw bones and almost universal absence in any other skeleton. Another school of thought suggests that histopathologically it shows stellate cells with branching processes in mucopolysaccharide background. Occasionally, macrophages and islands of inactive odontogenic epithelium were also identified. The cells are similar to myofibroblast, thus confirming OMs are of ectomesen-chymal origin.^5^

Differential diagnosis includes odontogenic cyst, ame-loblastoma, intraosseous hemangioma, and metastatic lesions, and each should be ruled out finally by means of histopathologic examination and immunohistochemistry.

Treatment of OMs varies depending on the extension of the lesion. Small tumors are usually treated by curet-tage, and for larger lesions, more extensive radical resection may be required.^6^ In this case, the lesion was large with an intact lower border in a developing mandible, so utmost care was taken while removing a lesion, thereby preventing pathological fracture.

Since OMs are bereft of a capsule and have an infiltra-tive growth pattern, there is a high rate of recurrence.^1^ Cryotherapy as a supplementary technique to curettage can be used to reduce this risk.^7^

Advanced imaging techniques, such as three-dimensional computed tomography (3D CT), magnetic resonance imaging (MRI), or cone-beam CT (CBCT) should be employed, to ensure the true extent of the tumor.^8^ They can be correlated with the histological features and are considered useful tools for diagnosis (internal structure of the lesion and the condition of bone margins).^9^

Histopathologically, the OM is distinguished by the presence of loose, abundant mucoid stroma that contains rounded, spindle-shaped, or stellate cells. The stroma may be relatively avascular or may exhibit delicate capillaries.^10^

A follow-up is necessary to confirm the healing and check the recurrence. Periodical clinical and radiographic screening should be maintained indefinitely irrespective of the treatment modality employed.^1^

## CONCLUSION

Odontogenic myxoma is considered as a tumor of undecided pathogenesis. They possess a notorious habit of recurrence. Odontogenic myxoma may exhibit variable features on imaging, when we use plain radiographs in different projection, and can be misleading for the clinicians. Further diagnostic 3D imaging techniques, such as CBCT, CT scans, and MRI, should be preferred to give more radiological details about the size and nature of lesion. It helps the clinicians in deciding whether conventional or radical intervention should be planned. The drawbacks of these diagnostic modalities are that they cannot be afforded by patients of low socioeconomic status and their selective availability, which are to be considered for better outcome.

## References

[1] Manne RK, Kumar VS, Venkata Sarath P, Anumula L, Mundlapudi S, Tanikonda R (2012). Odontogenic myxoma of the mandible.. Case Rep Dentistry.

[2] Kawase-Koga Y, Saijo H, Hoshi K, Takato T, Mori Y (2014). Surgical management of odontogenic myxoma: a case report and review of the literature.. BMC Res Notes.

[3] Zhang J, Wang H, He X, Niu Y, Li X (2007). Radiographic examination of 41 cases of odontogenic myxomas on the basis of conventional radiographs.. Dentomaxillofac Radiol..

[4] Rani V, Mahaboob Kadar M, Babu A, Sankari L, Krishnasamy G (2014). Odontogenic myxoma diagnostic dilemma: a case report and review of literature.. J Cranio-Maxillary Dis.

[5] Rajendran A, Sundaram S Shafer’s textbook of oral pathology.

[6] Hemavathy S, Vinay Kumar D (2014). Odontogenic myxoma. A case report.. IOSR J Dent Med Sci.

[7] Aquilino R, Tuji F, Eid N, Molina O, Joo H, Neto F (2006). Odonto-genic myxoma in the maxilla: a case report and characteristics on CT and MR.. Oral Oncol Extra.

[8] Spencer K, Smith A (1998). Odontogenic myxoma: case report with reconstructive considerations.. Aust Dent J.

[9] Deliverska E (2013). Odontogenic myxoma: a rare case and diagnostic and therapeutic challenges.. J IMAB.

[10] Reddy S, Naag A, Kashyap B (2010). Odontogenic myxoma: report of two cases.. Natl J Maxillofac Surg.

